# Regulation of Physical Microglia–Neuron Interactions by Fractalkine Signaling after Status Epilepticus

**DOI:** 10.1523/ENEURO.0209-16.2016

**Published:** 2017-01-16

**Authors:** Ukpong B. Eyo, Jiyun Peng, Madhuvika Murugan, Mingshu Mo, Almin Lalani, Ping Xie, Pingyi Xu, David J. Margolis, Long-Jun Wu

**Affiliations:** 1 Department of Cell Biology and Neuroscience, Rutgers University, Piscataway, NJ 08854; 2 Department of Neurology, Mayo Clinic, Rochester, MN 55905; 3 Department of Neurology, First Affiliated Hospital of Guangzhou Medical University Guangdong, 510120, China

**Keywords:** epilepsy, fractalkine, glutamate, interleukin-1β, microglia, seizure

## Abstract

Microglia, the resident immune cells of the brain, perform elaborate surveillance in which they physically interact with neuronal elements. A novel form of microglia–neuron interaction named microglial process convergence (MPC) toward neuronal axons and dendrites has recently been described. However, the molecular regulators and pathological relevance of MPC have not been explored. Here, using high-resolution two-photon imaging *in vivo* and *ex vivo*, we observed a dramatic increase in MPCs after kainic acid– or pilocarpine-induced experimental seizures that was reconstituted after glutamate treatment in slices from mice. Interestingly, a deficiency of the fractalkine receptor (CX3CR1) decreased MPCs, whereas fractalkine (CX3CL1) treatment increased MPCs, suggesting that fractalkine signaling is a critical regulator of these microglia–neuron interactions. Furthermore, we found that interleukin-1β was necessary and sufficient to trigger CX3CR1-dependent MPCs. Finally, we show that a deficiency in fractalkine signaling corresponds with increased seizure phenotypes. Together, our results identify the neuroglial CX3CL1–CX3CR1 communication axis as a modulator of potentially neuroprotective microglia–neuron physical interactions during conditions of neuronal hyperactivity.

## Significance Statement

Microglia, the immune cells of the brain, are exquisitely sensitive to disturbances in brain homeostasis and are critical for proper neuronal function. Seizures are a common disorder of the brain. However, the dynamics of microglial interactions with neurons after such conditions are not known. Using high-resolution real-time imaging in living mouse brain tissues, we have discovered an interesting phenomenon wherein brain microglia physically interact with neurons after hyperactive conditions. Specifically, we have elucidated relevant mechanisms and molecular signaling cascades governing these interactions. In addition, our findings suggest neuroprotective roles for microglial interaction with neurons. Together, our results suggest that enhancing microglial function during seizures may serve beneficial therapeutic functions.

## Introduction

Epilepsy is a significant health concern affecting 50–65 million people globally (Thurman et al., 2011) and is comorbid with stroke and traumatic brain injury (Temkin, 2009; Ravizza et al., 2011). Seizures in epilepsy are mainly due to abnormal hypersynchrony of neuronal activities (Fisher et al., 2005), which could be caused by an imbalance of excitatory and inhibitory neurotransmission (Dalby and Mody, 2001; Sharma et al., 2007). However, therapeutic antiepileptic strategies targeting neuronal mechanisms have proved insufficient in a significant number of patients (Kwan and Brodie, 2006). Therefore, there is a need to develop novel alternative and complementary strategies for seizure treatment.

Recently, a role for neuroinflammation has become increasingly appreciated in the pathological progression of epilepsy (Devinsky et al., 2013; Vezzani et al., 2013; Eyo et al., 2016). Microglia are the predominant source of inflammation in the brain and are now recognized to play significant roles in brain homoeostasis (Hanisch and Kettenmann, 2007; Ransohoff and Perry, 2009). Particularly, microglia make transient physical interactions with neuronal elements and, in doing so, are suggested to monitor and alter synaptic activity (Wake et al., 2009; Tremblay et al., 2010; Kato et al., 2016). Moreover, neuronal hyperactivity, including after status epilepticus, triggered increasing microglial process extension to interact with neurons (Dissing-Olesen et al., 2014; Eyo et al., 2014). Previous studies reported a novel microglia–neuron physical interaction named microglial process convergence (MPC) toward neuronal dendrites under conditions of reduced extracellular calcium (Eyo et al., 2015) as well as after repetitive neuronal stimulation (Kato et al., 2016). However, molecular regulators guiding MPC and whether they are functionally relevant in epilepsy remain to be elucidated.

Fractalkine (CX3CL1) is a neuronal chemokine, whose receptor (CX3CR1) is principally expressed by microglia in the CNS (Cardona et al., 2006). Given the distinct expression of fractalkine and its receptor in neurons and microglia, respectively, this communication axis presents a potentially unique avenue for microglia–neuron interactions. Indeed, fractalkine signaling was shown to regulate learning and memory (Maggi et al., 2011), social behaviors (Zhan et al., 2014), and microglial neurotoxicity during inflammation and stroke (Cardona et al., 2006; Tang et al., 2014). Under epileptic conditions, recent studies found that fractalkine deficiency resulted in reduced dendritic complexity and delayed maturation of adult newborn neurons (Xiao et al., 2015). However, the exact role of fractalkine signaling in seizure-induced microglia–neuron physical interactions has not been investigated. In the current study, we found that experimentally induced seizures triggered increased MPCs toward neuronal dendrites, which are regulated by fractalkine and interleukin (IL)-1β signaling. Moreover, reduced MPCs correlated with worsened seizure phenotypes. Our study reveals a novel regulation of microglia–neuron physical interaction by fractalkine signaling that is relevant during seizures.

## Materials and Methods

### Animals

Both male and female mice were used in accordance with institutional guidelines, as approved by the animal care and use committee at the university. Heterozygous (CX3CR1^GFP/+^) and homozygous (CX3CR1^GFP/GFP^) GFP reporter mice expressing GFP under control of the fractalkine receptor (CX3CR1) promoter (Jung et al., 2000) and transgenic mice expressing YFP (Feng et al., 2000), or a genetically encoded calcium sensor, i.e GCaMP2.2/GCaMP3.3 (Chen et al., 2012) in a subset of pyramidal neurons under the control of the Thy1 promoter were purchased from the Jackson Laboratory. The CX3CR1^GFP/GFP^ line as a knock-in GFP mouse line that serves as a CX3CR1 knockout mouse in this study. P2Y12 knockout mice were originally donated by Dr. Michael Dailey at the University of Iowa (Iowa City, IA).

### Slice preparation

Freshly isolated brain slices were prepared from 3- to 5-week-old mice. Briefly, mice were anesthetized and swiftly decapitated. Brains from decapitated mice were carefully removed and placed in ice-cold oxygenated (95% O_2_ and 5% CO_2_) artificial cerebrospinal fluid (ACSF) with the following composition (in mm): NaCl, 124; NaHCO_3_, 25; KCl, 2.5; NaH_2_PO_4_, 1; CaCl_2_, 2; MgSO_4_, 2; glucose, 10; and sucrose added to make 300–320 mOsmol. Coronal slices (300 µm) were prepared and transferred to a recovery chamber for ≥30 min with oxygenated ACSF with the same composition as above at room temperature before imaging.

### Preparation for *in vivo* imaging

Thirty- to sixty-day-old mice were anesthetized with isoflurane. We used 5% isoflurane for induction for up to 1 min in a chamber until the mouse was still, and 1.5%–2% for surgery for 15–30 min in the stereotactic frame. Under anesthesia, the mouse head was secured with ear bars on a heating pad and in a stereotactic frame, and a thin skull window was made with a high-speed dental drill. A head plate was glued to the skull around the cranial window, and the plate was screwed into a customized stage and placed under the two-photon microscope. Mice were maintained under light anesthesia (1% isoflurane) on the imaging stage for the duration of imaging and killed immediately after.

### Two-photon imaging

Experiments were conducted at room temperature with slices maintained in oxygenated ACSF with the same composition as above in a perfusion chamber at a flow rate of ∼2 mL/min. Microglia from heterozygous (CX3CR1^GFP/+^) and homozygous (CX3CR1^GFP/GFP^) GFP reporter mice expressing GFP under control of the fractalkine receptor (CX3CR1) promoter (Jung et al., 2000) and neurons from and transgenic mice expressing YFP (Feng et al., 2000), or a genetically encoded calcium sensor, i.e. GCaMP2.2/GCaMP3.3 (Chen et al., 2012) under the control of the Thy-1 promoter were typically imaged using a two-photon microscope (Scientifica) with a Ti:Sapphire laser (Mai Tai; Spectra Physics) tuned to 890–900 nm with a 40× water-immersion lens (0.8 NA; Olympus). Fluorescence was detected using two photomultiplier tubes in whole-field detection mode and a 565-nm dichroic mirror with 525-/50-nm (green channel) and 620-/60-nm (red channel) emission filters. The laser power was maintained at ≤25 mW, and images were collected at 50–120 µm from the slice surface, or at <40 mW, 50–120 µm of the cortical surface *in vivo*. For imaging microglial and neuronal YFP dynamics, 15 consecutive *z*-stack images were collected at 3-µm intervals every minute while 10 consecutive *z*-stack images were collected at 2-µm intervals every 30 s during imaging in GCaMP2.2 tissues. For *in vivo* imaging, 20 consecutive *z*-stack images were collected at 1.5-µm intervals every minute.

### Drugs

Glutamate and NMDA were purchased from Sigma. 6-Cyano-7-nitroquinoxaline-2,3-dione, kainic acid, tetrodotoxin (TTX), D-AP5, (+)-α-methyl-4-carboxyphenylglycine, and dihydroxyphenylglycine were purchased from Tocris. Recombinant mouse fractalkine (472-FF-02), IL-1β (401-ML), function-blocking fractalkine antibody (α-CX3CL1; MAB571), and IL-1ra (480-RM) were purchased from R&D Systems. Stock solutions of all drugs (except TTX) were made in water and diluted to the appropriate working concentrations in ACSF. TTX stock was diluted in citric acid (pH 4.8). The drugs were applied to the slices through a bath perfusion.

### Experimental seizure models

Thirty- to sixty-day-old mice were i.p. injected with kainic acid at 18–22 mg/kg or pilocarpine at 300 mg/kg. Seizure behavior was monitored under a modified Racine scale as follows: (1) freezing behavior; (2) rigid posture with raised tail; (3) continuous head bobbing and forepaws shaking; (4) rearing, falling, and jumping; (5) continuous occurrence of level 4; and (6) loss of posture and generalized convulsion activity (Racine, 1972; Avignone et al., 2008). Mice that progressed to at least stage 3 were killed at 2 h, and microglial dynamics were subsequently monitored in the slices generated. Alternatively, mice were used for microglial imaging *in vivo*. To block NMDA receptors, AP5 (3 µg in 5 µL ACSF) was applied through a previously implanted cannula for intracerebroventricular delivery 15 min before and 30 min after i.p. injection of kainic acid.

### Statistical analysis

Quantification of process convergence events was done manually through time-lapse movies. Events were identified when microglial processes spontaneously converged toward a focal point. These converging processes were redirected from normal random surveillance (extension/retraction) toward the focal region and terminated their convergence within minutes from one to four nearby microglia. This is best visualized in the movies presented (Movies 1–5). We noticed a variability in the sizes of the focal convergences from ∼2 to 8 µm. To avoid arbitrary selection of these events, analysis was done by counting all the observed events irrespective of size so as not to bias our analysis/quantification. At least five slices and two animals were used for each experimental condition. The frequency of occurrence of these events was determined in our typical 330 × 330 × 45-µm field of view from 30-min long imaging sessions in slices and 220 × 220 × 45-µm field of view from 60-min long imaging sessions *in vivo*. Data are presented as mean ± SEM. Student’s *t*-test was used to establish significance.

## Results

### Experimental seizures trigger microglial process convergence

**Figure 1.**
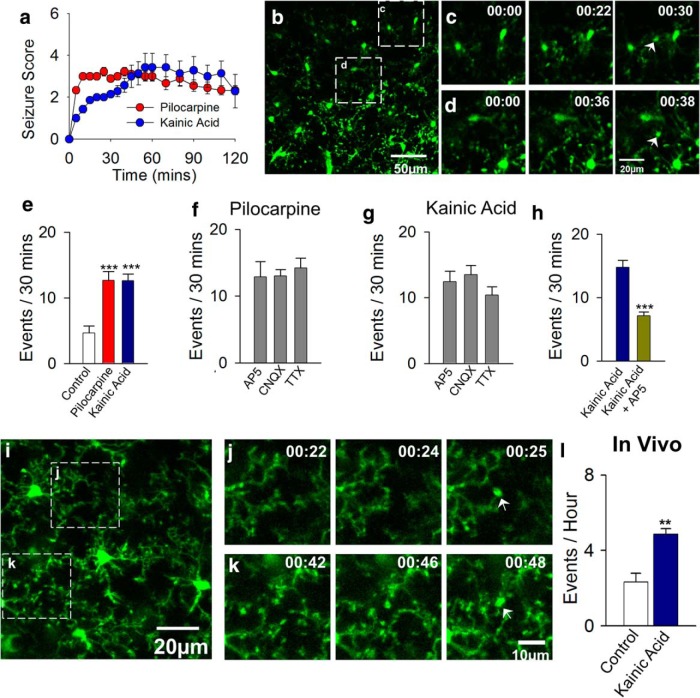
Experimental seizures trigger microglial process convergence (MPCs). ***a***, Seizure scores after intraperitoneal delivery of pilocarpine (300 mg/kg) and kainic acid (18–22 mg/kg). ***b–d***, Representative images of MPCs in acute slices from mice after kainic acid treatment. ***c***, ***d***, Time-lapse images of boxed regions in ***b*** showing converging microglial process foci identified with white arrows. ***e***, Quantitation of MPCs under control, pilocarpine, and kainic acid treatment (*n* = 9–14 slices each). ***f***, ***g***, Ionotropic glutamate receptor antagonists and action potential blockers fail to block pilocarpine- (***f***) or kainic acid– (***g***) induced MPCs (*n* = 5–9 slices each). ***h***, NMDA receptor antagonism during kainic acid–induced seizures reduces MPCs (*n* = 8–12 slices each). ***i–k***, Representative images of MPCs *in vivo* from mice after kainic acid treatment. ***j***, ***k***, Time-lapse images of boxed regions in ***i*** showing MPC foci identified with white arrows. ***l***, Quantification of MPCs *in vivo* after kainic acid–induced seizures (*n* = 4–5 mice each). **p < 0.01; ***p < 0.001.

The existence of a novel form of microglia–neuron physical interaction termed microglial process convergence (MPCs) was reported that increased upon extracellular calcium reduction (Eyo et al., 2015), a paradigm known to lead to epileptiform burst activity (Bikson et al., 1999) and prolonged neuronal depolarization (Kato et al., 2016). Typically, MPCs consist of several microglial processes that spontaneously converge at distinct sites and make transient focal aggregations (see movies). To determine whether these phenomena are present during epileptic conditions, we induced experimental seizures (at least stage 3 seizures along a modified Racine scale) by intraperitoneal injection of kainic acid (18–22 mg/kg) or pilocarpine (300 mg/kg; Fig. 1*a*). Two hours after seizure induction, brain slices were generated from mice and monitored for MPC events by time-lapse two-photon microscopy. We detected a significant number of these events in real time in cortical slices after both kainic acid– and pilocarpine-induced seizures (Fig. 1*b–e*; Movie 1). The MPC events were maintained independently of ionotropic glutamate receptor function and action potential firing (Fig. 1*f*, *g*). However, when NMDA receptors were antagonized during seizures by a 15-min pre- and 30-min posttreatment of AP5 (3 µg in 5 µL ACSF) with kainic acid treatment, the number of MPCs was significantly reduced (Fig. 1*h*), indicating that NMDA receptors are required for the induction of seizure-induced MPC events. To exclude the possibility that seizure-induced MPCs occur because of the brain slice preparation, we used *in vivo* two-photon microscopy to monitor microglial dynamics in the intact cortex after 2 h of kainic acid treatment. Consistent with our observations in brain slices, we found a significant increase in the occurrence of MPCs *in vivo* after kainic acid treatment (Fig. 1*i–l*).

Movie 1. Microglial process convergence occurs after seizures. Representative time-lapse movies taken from CX3CR1^GFP/+^ mouse slices showing MPCs events. Slices were generated from the brains of mice 2 h after kainic acid treatment. Several MPCs events are identified (white arrows). This movie is 140 min long and is sped up 120×. 10.1523/ENEURO.0209-16.2016.video.1

### Transient glutamate treatment mimics seizure-induced MPCs

**Figure 2.**
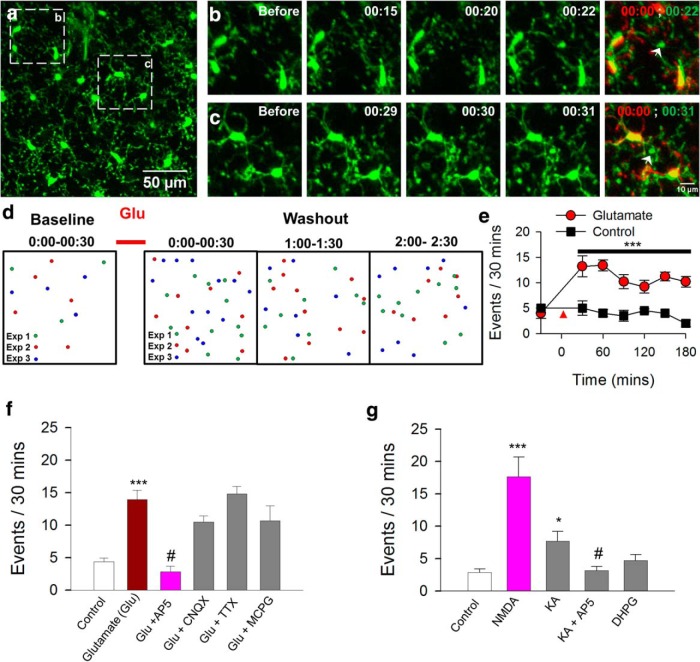
Transient glutamate treatment mimics seizure-induced MPCs. ***a–c***, Images from a time-lapse sequence showing converging microglial processes in a slice after glutamate (10 mm) treatment for 10 min. ***b***, ***c***, Time-lapse images of boxed regions in ***a*** showing converging microglial processes foci identified with white arrows. ***d***, ***e***, Schematic of sites of process convergence events (***d***) and quantified summary (***e***) showing increased occurrence after glutamate treatment (red arrowhead in ***e***, *n* = 3 slices each). ***f***, ***g***, Quantification of the number of process convergence events under different conditions showing a role for NMDA receptors (*n* = 5–14 slices each). *p < 0.05; ***p < 0.001 compared with control and #*p* < 0.01 compared with previous condition in the respective graphs.

Seizures are known to result from an increase in neuronal excitation that corresponds with an increase in glutamate release in the brain (Cavus et al., 2005, 2008). To simulate and study the mechanisms underlying seizure-induced MPCs, we treated slices from naive mice with glutamate (1 mm) to mimic seizure activities. Interestingly, whereas vehicle treatment did not increase the occurrence of MPCs, 10 min of glutamate treatment was able to significantly increase MPC events (Fig. 2*a–c*; Movie 2), and this increase persisted for up to 3 h after treatment (Fig. 2*d*, *e*).

Movie 2. Glutamate induces MPCs. Representative time-lapse movie taken from slices excised from a CX3CR1^GFP/+^ mouse previously exposed to 10 min of 1 mm glutamate. Three MPC events can be seen in different regions of the slice and are identified with white arrows. This movie is 75 min long and is sped up 180×. 10.1523/ENEURO.0209-16.2016.video.2

Consistent with seizure-induced MPCs, glutamate-induced MPCs required NMDA receptor activation for their induction, since they were blocked by coapplication of AP5 (50 µm) with glutamate (Fig. 2*f*). However, antagonists for non-NMDA glutamate receptors (6-cyano-7-nitroquinoxaline-2,3-dione, 10 µm), voltage-gated sodium channels (TTX, 1 µm), and metabotropic glutamate receptors [(+)-α-methyl-4-carboxyphenylglycine, 200 µm] could not inhibit glutamate-induced MPCs (Fig. 2*f*). Moreover, activation of the NMDA receptor was sufficient to trigger MPCs, whereas activation of non-NMDA glutamate receptors (by kainic acid) failed to induce MPCs without NMDA receptors (Fig. 2*g*).

**Figure 3.**
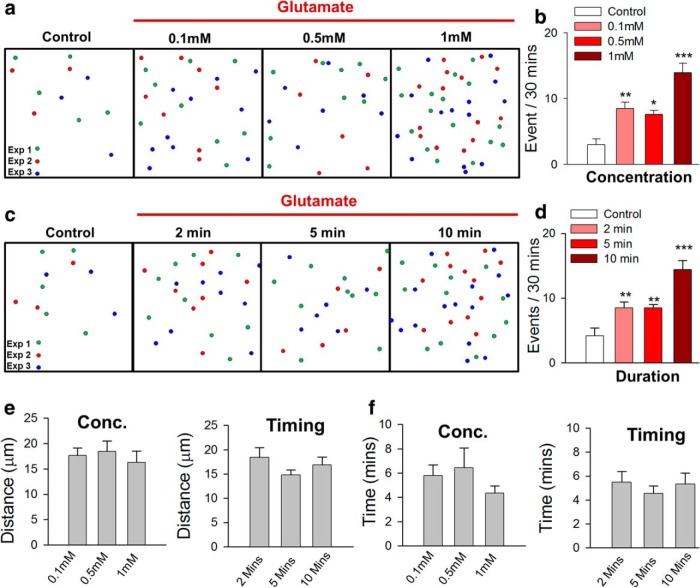
Characteristics of glutamate-induced MPCs. ***a–d***, Schematic representation (***a***, ***c*)** and quantitative summary (***b***, ***d***) showing the occurrence of MPC events at various glutamate concentrations for 10 min (***a***, ***b*)** and various time points at 1 mm (***c***, ***d*)**. *n* = 6–11 slices each***. e***, ***f***, Microglia respond from similar distances in all conditions of glutamate exposure tested. Glutamate-induced MPCs show similar features in the different conditions. **p < 0.01; ***p < 0.001 compared with control.

**Figure 4.**
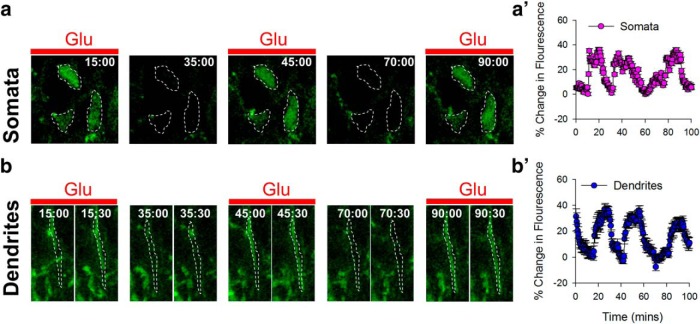
Neurons respond functionally to multiple hits of glutamate. Images (***a***, ***b*)** and time series (***a′***, ***b′*)** data showing intracellular calcium transients in neuronal somata (top) and dendrites (bottom) from GCaMP2.2 mouse slices after repeated glutamate (1 mm) treatment in cortical layer II/III.

Although 1 mm glutamate (10-min) treatment yielded robust increases in MPCs, lower concentrations (0.1–0.5 mm) for the same duration or 1 mm glutamate for shorter durations (2–5 min) were also able to increase MPC numbers significantly, although less robustly (Fig. 3*a–d*). In these different conditions, MPCs exhibited similar features with regard to the maximum distance from which they responded and the time to complete the convergence (Fig. 3*e*, *f*). Finally, because glutamate can lead to excitotoxicity, we confirmed via calcium imaging in Thy1-GCaMP3.3 and Thy1-GCaMP2.2 mice that after a 10-min, 1-mm glutamate treatment, cortical neurons continued to exhibit somatic and dendritic calcium responses, suggesting that the initial glutamate treatment did not render these cortical neurons functionally unresponsive (Fig. 4).

**Figure 5.**
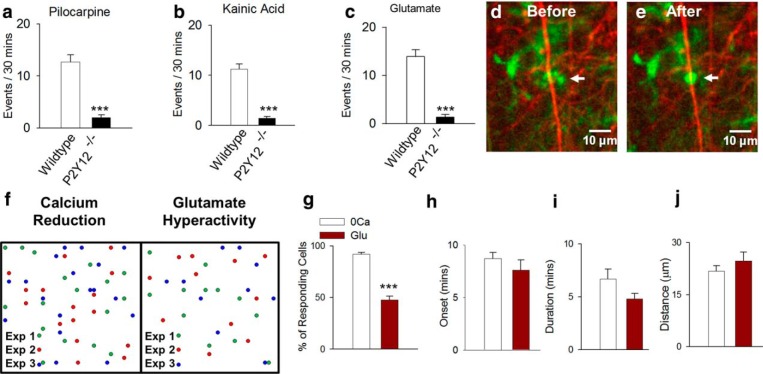
Comparison between calcium reduction–induced and glutamate-induced MPCs. ***a–c***, Quantitative summary of MPC events in slices from wild-type and P2Y12-deficient mice after pilocarpine-induced seizures (***a***), kainic acid–induced seizures (***b***) and 10-min glutamate treatment (***c***) (*n* = 3–7 slices each). ***d***, Example of a converging microglial (green) process (arrow) terminating on a neuronal dendrite (red) after glutamate treatment in a slice from a double transgenic CX3CR1^GFP/+^:Thy1^YFP/+^ mouse. ***f***, ***g***, Schematic representation of sites of convergence foci (***f*)** and quantitative summary data (***g***) showing sites and number of cells that respond during the different treatments. ***h–j***, Various features of MPCs are similar between the two methods of convergence (*n* = 6–12 slices each). ***p < 0.001.

We confirmed that both seizure- and glutamate-induced MPCs require P2Y12 receptors (Fig. 5*a–c*), similar to MPC during [Ca^2+^]_o_ reduction (Eyo et al., 2015). Furthermore, we confirmed that the glutamate-induced events terminated on neuronal dendrites like the [Ca^2+^]_o_ reduction-induced events (Eyo et al., 2015; Fig. 5*d*, *e*; Movie 3). We next compared several characteristics of [Ca^2+^]_o_ reduction– and glutamate-induced MPCs and found MPCs are more robust when induced by [Ca^2+^]_o_ reduction, with >90% of microglia exhibiting MPCs in a 30-min time frame compared with only ∼50% of microglia after glutamate treatment (Fig. 5*f*, *g*). However, other features were similar between the two induction methods, including onset time, duration of completed contact, and distance from which responding cells completed contact (Fig. 5*h–j*). Together, these results highlight a distinction between the occurrence of glutamate-induced and [Ca^2+^]_o_ reduction–induced MPCs.

Movie 3. Glutamate-induced MPCs targets neuronal dendrites. Representative time-lapse movie taken from slices excised from a CX3CR1^GFP/+^;Thy1^YFP/+^ mouse previously exposed to 10 min of glutamate. A microglial process (green) convergence event terminating on a labeled dendrite (red) is identified with a white arrow. This movie is 48 min long and is sped up 240×. 10.1523/ENEURO.0209-16.2016.video.3

Because astrocytes can also release ATP (Ben Achour and Pascual, 2012; Torres et al., 2012) to mediate MPCs, we investigated contributions by astrocyte connexins to MPC induction with carbenoxolone (100 µm). MPC numbers in response to glutamate treatment remained unchanged in the presence of carbenoxolone (*n* = 8; 13.5 ± 2.4 events per 30 min) compared to in its absence (*n* = 6; 12.1 ± 1.2 events per 30 min). Moreover, wholesale inhibition of astrocyte metabolism by fluoroacetate (2–10 mM), which we have found reduces spontaneous astrocyte calcium transients (Eyo et al., 2015), did not change MPC numbers (12.7 ± 1.8 events per 30 min without fluoroacetate compared to 11.9 ± 1.2 events per 30 min with fluoroacetate; *n* = 7–8). Together, these data suggest that astrocytes might not directly participate in glutamate-induced MPCs.

### Fractalkine signaling is necessary and sufficient to trigger MPCs

**Figure 6.**
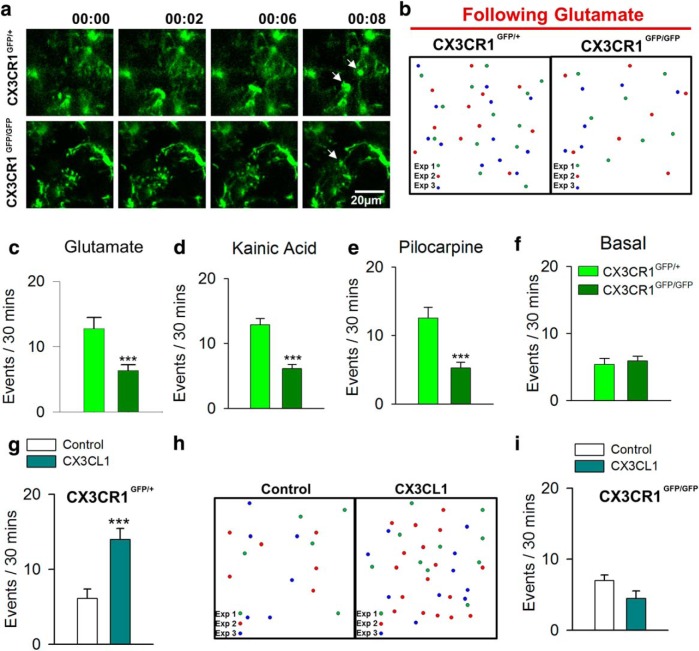
Fractalkine signaling is necessary and sufficient to trigger MPCs. ***a***, ***b***, Images from a time-lapse sequence (***a***) and summary schematic (***b***) showing converging microglial processes in slices from CX3CR1^GFP/+^ and CX3CR1^GFP/GFP^ after glutamate treatment. ***c–f***, Quantification of MPC numbers in slices from CX3CR1^GFP/+^ and CX3CR1^GFP/GFP^ after glutamate treatment (***c***), kainic acid–induced seizures (***d***), and pilocarpine-induced seizures (***e***) showing reduced events in CX3CR1^GFP/GFP^ slices as well as under basal conditions (***f***; *n* = 7–13 slices each). ***g–i***, Quantified summary of MPC numbers after CX3CL1 (200 ng/mL) application in slices from CX3CR1^GFP/+^ (***g***, ***h***) and CX3CR1^GFP/GFP^ (***i***) mice (*n* = 6–8 slices each). ***p < 0.001.

To determine a molecular regulator of seizure- and glutamate-induced MPCs, we turned to fractalkine (CX3CL1-CX3CR1) signaling, which has been shown to be a paramount signaling mechanism for microglia–neuron interactions (Paolicelli et al., 2014). To this end, we performed imaging in slices generated after either glutamate treatment or experimental seizures induced by kainic acid or pilocarpine from CX3CR1^GFP/+^ and CX3CR1^GFP/GFP^ mice. Interestingly, we found a significant reduction in the number of MPC events in CX3CR1^GFP/GFP^ mice compared with CX3CR1^GFP/+^ mice (Fig. 6*a–e*; Movie 4), although the number of MPC events in basal conditions was not different between the genotypes (Fig 6*f*). In addition, this reduction in the number of MPCs did not result from a difference in the number of microglia in our imaging fields of view between genotypes (38.9 ± 1.0 cells per field of view in CX3CR1^GFP/+^ slices compared to 38.1 ± 1.3 cells per field of view in CX3CR1^GFP/GFP^ slices) or in various features of the convergence events such as the time to complete the convergence (data not shown). Next, we asked whether exogenous fractalkine (CX3CL1) is sufficient to induce MPCs in cortical slices. Indeed, we found that bath application of CX3CL1 (200 ng/mL) increased MPC occurrence in slices from CX3CR1^GFP/+^ mice (Fig. 6*g*, *h*) but failed to do so in slices from CX3CR1^GFP/GFP^ mice (Fig. 6*i*). Together, these results indicate that fractalkine signaling is sufficient for MPC induction and is required for glutamate- and seizure-induced MPCs.

Movie 4. Microglial process convergence in fractalkine receptor heterozygote (left) and knockout (right) slices after glutamate treatment. Representative time-lapse movie taken from CX3CR1^GFP/+^ (i.e., CX3CR1^+/–^, left) and CX3CR1^GFP/GFP^ (i.e., CX3CR1^–/–^, right) slices showing several MPC events (arrows) after a 10-min glutamate (1 mm) treatment. This movie is 30 min long and is sped up 180×. 10.1523/ENEURO.0209-16.2016.video.4

### Glutamate-induced MPCs requires IL-1β

**Figure 7.**
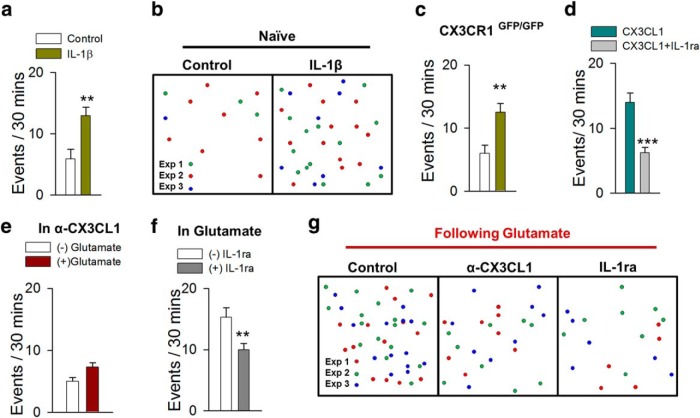
Glutamate-induced MPCs requires IL-1β. ***a–c***, Quantified summary (***a***, ***c***) and schematic representation (***b***) showing that IL-1β (30 ng/mL) increases MPCs in slices from both CX3CR1^GFP/+^ and CX3CR1^GFP/GFP^ mice (*n* = 6–17 slices). ***d***, CX3CL1-induced MPCs is blocked by IL-1ra (100 ng/mL), an IL-1β antagonist, in slices from CX3CR1^GFP/+^ mice (*n* = 8–9 slices each). ***e–g***, Glutamate-induced MPCs fails to occur in the presence of function-blocking CX3CL1 antibodies and is reduced in the presence of IL-1ra (*n* = 10–12 slices each). **p < 0.01; ***p < 0.001.

We attempted to determine factors downstream of fractalkine signaling that may directly induce MPCs. Because of the widespread evidence of fractalkine signaling regulating IL-1β (Cardona et al., 2006; Dénes et al., 2008; Rogers et al., 2011), we investigated its role in MPCs. Interestingly, we found that application of 30 ng/mL recombinant mouse IL-1β significantly increased MPCs in slices (Fig. 7*a*, *b*; Movie 5). This increase occurred independently of action potential firing, as the number of events were similar with IL-1β only (*n* = 6; 16.5 ± 1.4 events per 30 min) or IL-1β pretreated (30 min) and cotreated with TTX (1 µm; *n* = 8; 14.0 ± 1.4 events per 30 min). In addition, IL-1β also induced a significant increase of MPC events from mice deficient in CX3CR1 (CX3CR1^GFP/GFP^; Fig. 7*c*). To determine whether IL-1β functions downstream of CX3CL1-induced MPC (Fig. 6), we applied CX3CL1 with IL-1ra (100 ng/mL) to antagonize IL-1β function. In these experiments, IL-1ra significantly reduced the occurrence of MPCs (Fig. 7*d*). Finally, to confirm roles for fractalkine and IL-1β signaling in glutamate-induced MPCs, we performed glutamate experiments in the presence of either fractalkine neutralizing antibody (α-CX3CL1) or IL-1ra. Consistent with the foregoing results, glutamate failed to significantly increase MPCs in the presence of α-CX3CL1 (Fig. 7*e*, *g*) or IL-1ra (Fig. 7*f*, *g*). Together, these results indicate that glutamate-induced MPCs requires fractalkine and IL-1β signaling.

Movie 5. IL-1β increases MPCs. Representative time-lapse movie taken from CX3CR1^GFP/+^ showing that IL-1β (30 ng/mL) increases the occurrence of MPCs (arrows). This movie is 70 min long and is sped up 180×. 10.1523/ENEURO.0209-16.2016.video.5

### Neuroprotective potential of MCP

**Figure 8.**
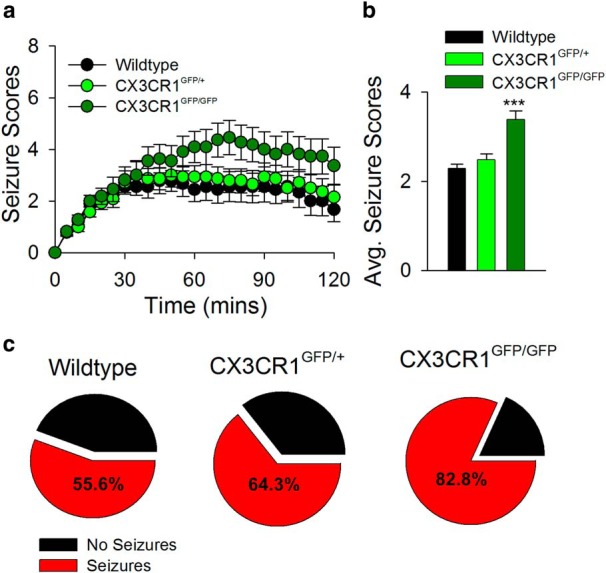
Neuroprotective potential of MPCs. ***a***, ***b***, Behavioral seizure scores in aged-matched wild type (*n* = 7), CX3CR1^GFP/+^ (*n* = 11), and CX3CR1^GFP/GFP^ (*n* = 9) mice treated with kainic acid through time (***a***) and on average (***b***). ***c***, Pie chart showing the percentage of mice from the seizure experiments that progressed to at least stage 3 seizures (red) along the Racine scale during the 2 h of seizure monitoring after kainic acid treatment. ***p < 0.001.

Our results indicate that MPCs generated after neuronal hyperactivity were promoted by fractalkine (Fig. 7) and purinergic (Fig. 5) signaling. To gain insights into the potential functional significance of these observations, we investigated the consequence of a deficiency of fractalkine signaling on acute seizures. It has previously been documented that in P2Y12^–/–^ mice, kainic acid treatment resulted in worsened seizure phenotypes (Eyo et al., 2014). Interestingly, we now report that a deficiency in the CX3CR1 receptor similarly resulted in increased seizure behaviors in response to kainic acid treatment (Fig. 8*a*, *b*). In addition, at the concentrations used, 55.6% of wild-type, 64.3% of CX3CR1^GFP/+^, and 82.8% of CX3CR1^GFP/GFP^ mice seized up to at least stage 3 on the modified Racine scale after kainic acid treatment (Fig. 8*c*). Moreover, whereas none of the wild-type and only 7.1% of the CX3CR1^GFP/+^ mice died, 27.3% of the CX3CR1^GFP/GFP^ mice died within the first 2 h of kainic acid treatment. These results indicate that a CX3CR1 deficiency results in increased seizure phenotypes and animal mortality that correlates with decreased MPCs.

## Discussion

**Figure 9.**
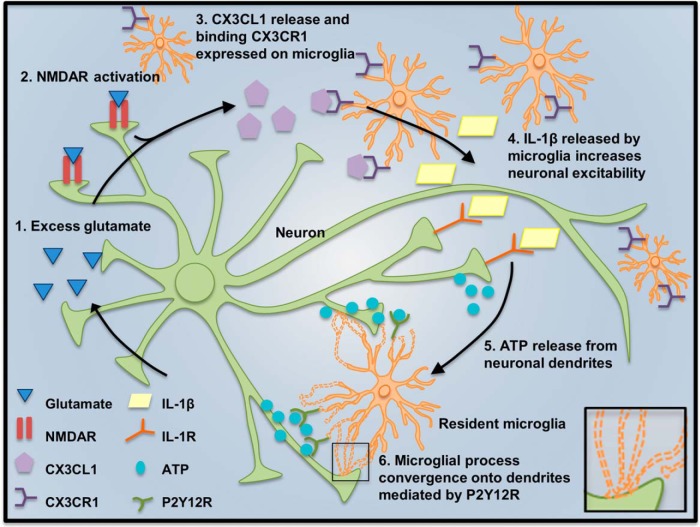
Model for glutamate-induced MPCs by fractalkine receptor–dependent signaling through IL-1β. Our results suggest a bidirectional mechanism of MPC in which (1) excessive glutamate release (2) activates neuronal NMDA receptors, which results in (3) the release of fractalkine from neuronal membranes that activates microglial fractalkine receptors. (4) Fractalkine receptor activation subsequently triggers IL-1β release from microglia, which in turn increases neuronal excitability to elicit (5) a localized release of ATP at specific dendritic hotspots. Finally, (6) ATP released from these hotspots attracts microglial processes via P2Y12 receptor to converge at the release site.

In the current study, we investigated the real-time dynamics of microglia–neuron interactions after experimental seizures and glutamate-induced hyperactivity using two-photon microscopy. We extend the findings of previous studies and show that, in addition to depleted extracellular calcium conditions and repeated neuronal stimulation, MPCs are induced in experimental seizure models and by glutamate application. We further delved into the mechanism of MPC induction and show that NMDA receptors are required to trigger these interactions. Furthermore, we determined a molecular regulation of the MPC phenomena by fractalkine signaling through IL-1β release (Fig. 9). Finally, we correlated the CX3CR1-dependent role in MPC generation with neuroprotection during acute seizures. Our results suggest a neuroprotective bidirectional microglial–neuronal communication axis after status epilepticus and provide novel evidence for microglial–neuronal physical interactions in acute epilepsy in the brain.

### Microglial process convergence: a distinct form of microglial–neuronal physical interactions

In this study, we report MPCs, a novel form of microglia–neuron physical interactions, that are dramatically increased after status epilepticus. It is important to note that the seizure-induced MPC event described here is distinct from the transient process extension phenomena previously reported (Dissing-Olesen et al., 2014; Eyo et al., 2014), even though both are NMDA and P2Y12 dependent. They are different in that although (a) process extension is exhibited by all processes (global) at a given time point after glutamate/NMDA treatment, MPCs are exhibited by select processes at a given time point in a focalized (local) manner, (b) process extension occurs only immediately after glutamate/NMDA treatment, MPCs persist for hours even after the withdrawal of the inducing agent, and (c) process extension is mechanistically independent of fractalkine signaling, whereas MPCs require it. These and other considerations indicate that MPCs represent a novel form of microglia–neuron physical interactions distinct from NMDA-dependent microglial process extension.

More importantly, MPCs triggered during hyperactive neuronal conditions as reported here are also distinct from MPCs previously reported under reduced [Ca^2+^]_o_ conditions (Eyo et al., 2015) and repeated neuronal depolarization (Kato et al., 2016) for several reasons. First, MPCs under reduced [Ca^2+^]_o_ conditions do not require NMDA receptors or action potentials, as they are not blocked with AP5 treatment (data not shown). Although low extracellular calcium levels are known to occur in epileptic contexts (Wadman et al., 1985; Heinemann et al., 1986; Konnerth et al., 1986), removal of extracellular calcium or doubling the extracellular calcium concentration did not alter the occurrence of MPCs in response to glutamate (data not shown), suggesting that glutamate-induced MPCs are not regulated by extracellular calcium concentrations. Similarly, MPCs after repeated neuronal stimulation required action potentials (Kato et al., 2016), whereas seizure-induced MPCs did not. Second, MPCs under reduced [Ca^2+^]_o_ conditions are not modulated by fractalkine signaling, as they are unaltered in CX3CR1^GFP/GFP^ mice. Third, MPCs elicited after repeated neuronal stimulation are directed toward axons, whereas the reported seizure-induced MPCs in our study are directed toward dendrites. However, because the various phenomena require microglial P2Y12 receptors, it is likely that the localized ATP release is similar under the different conditions. However, it remains to be determined whether common mechanisms are used to release ATP from neuronal dendrites in reduced [Ca^2+^]_o_ conditions, repeated neuronal stimulation, and after status epilepticus. Together, these considerations indicate that we have uncovered a unique form of microglia–neuron physical interactions in response to seizure activities in the brain.

### The mechanism of MPCs

Microglial interactions with neuronal elements in real time have been widely evidenced (Wake et al., 2009; Tremblay et al., 2010; Li et al., 2012; Dissing-Olesen et al., 2014; Eyo et al., 2014, 2015; Kato et al., 2016). However, factors that modulate such interactions have not been identified. Over the last decade, a wealth of data has shown important roles for fractalkine signaling between microglia and neurons in the developing and mature CNS (Limatola and Ransohoff, 2014; Paolicelli et al., 2014), but this unique signaling axis has not been sufficiently interrogated with regard to microglia–neuron physical interactions. Here, we found that fractalkine signaling is critical for this interaction, showing reduced MPCs in CX3CR1-deficient tissues after glutamate application and in two seizure models. Although fractalkine signaling did not play a significant role in microglial process outgrowth to neuronal NMDA receptor activation (Dissing-Olesen et al., 2014) or microglial interactions with the axon initial segment of neurons (Baalman et al., 2015), it would be of interest to determine whether fractalkine signaling regulation is also present in previously described interactions such as the bulbous presynaptic, postsynaptic, and somata contacts of neurons by microglial processes (Wake et al., 2009; Tremblay et al., 2010; Li et al., 2012).

Our results suggest the following bidirectional mechanism for MPCs on to neuronal dendrites: after increased glutamate release, (1) neuronal NMDARs activation results in (2) the release of CX3CL1 from neuronal membranes that (3) activates microglial CX3CR1. CX3CR1 activation subsequently (4) triggers IL-1β release (presumably from microglia), which in turn (5) acts on neuronal dendrites triggering localized release of ATP that then elicits the localized convergence of microglial processes through P2Y12 receptors (Fig. 9). Consistent with our results, previous studies reported that cultured cortical neurons release CX3CL1 after an acute transient exposure to glutamate in an NMDAR-dependent manner (Chapman et al., 2000; Noda et al., 2011), and CX3CL1 treatment increases IL-1β levels in CNS tissues (Johnston et al., 2004; Clark et al., 2015). These lines of evidence strongly imply a role for fractalkine signaling–dependent release of IL-1β in seizure-/glutamate-induced MPCs. IL-1β, itself, is known to trigger ATP release when applied to slices (Sperlágh et al., 2004), and seizures increase IL-1β levels (Avignone et al., 2008), with microglia as the primary cells secreting IL-1β early during seizures (Eriksson et al., 1999; Vezzani et al., 1999, 2008b). Moreover, this release has been linked, at least in part, to a prior NMDAR activation (Eriksson et al., 2000; Jander et al., 2000). Presumably, the mechanism of action for IL-1β occurs through activating its receptors, which are predominantly expressed by neurons in the naive CNS (Ban et al., 1991; Ban, 1994). Consistent with our model, IL-1β has been shown to enhance neuronal excitability (Vezzani et al., 2008a; Vezzani and Viviani, 2014). Moreover, IL-1β depresses GABAergic neurotransmission (Wang et al., 2000), indirectly enhancing glutamatergic neurotransmission. Thus, IL-1β may induce MPCs by concomitantly enhancing NMDAR function and inhibiting GABAergic neuronal inhibition, resulting in an overall increase in neuronal excitability. Alternatively, IL-1β may trigger the opening of as-yet-unidentified channels through which ATP may be released in a localized fashion to mediate the defined convergence. Although the precise mechanism by which IL-1β acting on neurons would trigger ATP release remains to be determined, ATP and ADP are widely recognized to mediate microglial process chemotaxis through P2Y12Rs (Honda et al., 2001; Davalos et al., 2005; Wu et al., 2007).

### The pathological relevance of MPCs

Because glutamate transporters efficiently limit the extracellular concentration of glutamate, it is unlikely that our findings are relevant for healthy brain conditions. Consistent with this fact, we detected a low MPC frequency in both naive brain slices and the intact cortex *in vivo*. However, because we found that the phenomenon could be elicited by shorter durations and lower concentrations of glutamate, we cannot rule out the possibility that MPCs could be triggered in the healthy brain during periods of intense physiological activity. In any case, our data suggest that the phenomenon is most relevant for conditions in which there is (even transient) increase in extracellular glutamate levels. Localized puff applications of glutamate did not reliably elicit MPCs (data not shown), suggesting that more global (rather than local) alterations in glutamate-dependent network activity are required for MPC induction. Indeed, we were able to observe an increase in MPCs in both brain slices and *in vivo* after experimental seizures, when global synchronized increases in neuronal network activities are known to occur.

In summary, we report here the existence of a novel microglia–neuron physical interaction phenomenon, microglial process convergence, or MPCs, that occurs after elevated glutamate levels in the murine cortex and is relevant during epileptic pathologies. Furthermore, we have determined some of the key players in the mechanism underlying the bidirectional communication between microglia and neurons, such as fractalkine signaling, IL-1β release, and P2Y12R-induced chemotaxis. Our results show a correlation between reduced MPCs (Figs. 5 and 7) and increased seizure severity and animal mortality with genetic depletion of microglial P2Y12 receptors (Eyo et al., 2014) and CX3CR1 receptors (Fig. 8). Although this relationship is not clearly causal between the two phenomena, it is suggestive of a neuroprotective relationship between MPCs and seizure consequences.

## Synthesis

Reviewing Editor: Christian Lüscher, University of Geneva Decisions are customarily a result of the Reviewing Editor and the peer reviewers coming together and discussing their recommendations until a consensus is reached. When revisions are invited, a fact-based synthesis statement explaining their decision and outlining what is needed to prepare a revision will be listed below. The following reviewer(s) agreed to reveal their identity: Alfonso Araque.

Authors have satisfactorily addressed the concerns raised about the previous version.

I have no further comments.
